# OXTR DNA methylation differentiates men on the obesity spectrum with and without binge eating disorder

**DOI:** 10.1186/s13148-022-01318-3

**Published:** 2022-08-30

**Authors:** Katrin Elisabeth Giel, Kathrin Schag, Elisabeth Johanna Leehr, Isabelle Mack, Lea-Sarah Schuster, Ariane Wiegand, Stephan Zipfel, Manfred Hallschmid, Vanessa Nieratschker

**Affiliations:** 1 Department of Psychosomatic Medicine and Psychotherapy, Medical University Hospital Tübingen, Osianderstr. 5, 72076 Tübingen, Germany; 2 Centre of Excellence for Eating Disorders (KOMET), Osianderstr. 5, 72076 Tübingen, Germany; 3 Institute for Translational Psychiatry, University of Münster, Albert-Schweitzer-Straße 11, 48149 Münster, Germany; 4 Department of Psychiatry and Psychotherapy, Medical University Hospital Tübingen, Calwerstraße 14, 72076 Tübingen, Germany; 5 Institute of Medical Psychology and Behavioural Neurobiology, University of Tübingen, Otfried-Müller-Straße 25, 72076 Tübingen, Germany; 6 German Center for Diabetes Research (DZD), Otfried-Müller-Straße 10, 72076 Tübingen, Germany; 7 Institute for Diabetes Research and Metabolic Diseases of the Helmholtz Center Munich at the University of Tübingen (IDM), Otfried-Müller-Straße 10, 72076 Tübingen, Germany

**Keywords:** DNA, Eating disorder, Epigenetics, Methylation, Obesity, Oxytocin

## Abstract

**Background:**

The neuropeptide oxytocin (OXT) plays a role in the regulation of eating behavior and metabolism. OXT functioning is altered in patients with eating and weight disorders, and a variant of the oxytocin receptor gene (OXTR) has been associated with impulsive eating behavior as it is seen in patients with binge eating disorder (BED). Gene × environment interactions could play a role in BED. One mechanism mediating this interaction is the epigenetic alteration of gene expression. We therefore investigated if DNA methylation of the *OXTR* differs between individuals with obesity depending on a comorbid BED. We analyzed DNA methylation of the *OXTR* in peripheral blood of 227 individuals on the obesity spectrum (mean age: 40.3 ± 13.1 yrs; mean BMI: 38.6 ± 7.3 kg/m^2^), 130 of which were diagnosed with BED.

**Results:**

There were no overall differences in *OXTR* methylation between participants with and those without BED (*p* > 0.05), while both subgroups were comparable regarding age and body mass index (BMI), but significantly differed in sex distribution (*p* = 0.035). We found no relationship between mean DNA methylation and BMI or self-reported eating disorder (ED) pathology. Analyzing potential sex differences revealed a significantly lower *OXTR* DNA methylation in male participants with BED as compared to those without BED (*p* = 0.017). No such difference was found in the female subsample (*p* > 0.05).

**Conclusions:**

Clinically significant binge eating pathology might be associated with lower *OXTR* DNA methylation exclusively in males. The differential DNA methylation of *OXTR* in males with BED supports the view that BED represents a phenotype within the obesity spectrum that is characterized by specific vulnerability factors. A better understanding of the epigenetic underpinnings of the OXT system might contribute to the refinement of OXT administration approaches as potential interventions in eating and weight disorders.

## Background

Binge eating disorder (BED) is the most common eating disorder (ED) in the general population [1]. BED is characterized by recurrent binge eating episodes with subjective loss of control [2], and in contrast to bulimia nervosa (BN), no regularly compensatory behaviors are shown [3]. Thus, affected patients often develop overweight or obesity, and there is a significant overlap between obesity and BED [4]. Patients with BED could therefore constitute a phenotype within the obesity spectrum that is characterized by increased impulsivity [2, 5, 6]. Obesity is one of the most serious global health problems, and it has a complex multifactorial etiology [7]. It has been outlined that the large group of people affected by obesity is characterized by considerable heterogeneity and that it will be important to identify subgroups in order to develop better and more tailored interventions [7]. Patients with BED could constitute such a subgroup within the obesity spectrum [2]; hence, the approach of the present study is to compare individuals on the obesity spectrum with and without a BED diagnosis.

One interesting neuromodulatory system in the context of BED is the oxytonergic system as the regulation of the neuropeptide oxytocin (OXT) is thought to play a role in a broad spectrum of mental disorders [8]. While OXT has initially been primarily known for its role in social relationships and bonding, it turned out that it also directly or indirectly regulates a range of other psychological and physiological functions, including eating behavior and metabolism [9, 10]. Alterations in OXT function have been reported for different populations with eating and weight disorders [11, 12], while most evidence is available for patients with anorexia nervosa (AN) [11, 13, 14]. Yet, there is a scarcity of studies looking at OXT function in relation to undercontrolled eating patterns and across the overweight spectrum [11], including patients with obesity and BED. First evidence points to differential effects of OXT administration on food intake in obesity as compared to normal-weight males [15]. Thus, alterations in the oxytonergic system could also contribute to the BED phenotype and might constitute a delimitation from obesity without BED.

Indeed, preliminary genetic evidence from a large general population-based cohort and from patients with BN suggests a possible genetic risk for BED/BN-type eating disorders associated with the rs53567 variant of the oxytocin receptor gene (*OXTR*) [16, 17]. However, studies on the role of the *OXTR* gene in obesity are scarce [18]. As in other complex mental disorders, an interaction between genetic risk factors and environmental influences could play a significant role in moderating the susceptibility to EDs [19]. Along those lines, a recent study on childhood obesity impressively illustrates the influence of gene × environment (G × E) interactions within the *OXTR* [18]: Carriers of the A allele of the *OXTR* rs53576 variant were especially affected by obesity when they grew up in lower socioeconomic background families, but were less affected when their families were characterized by a higher socioeconomic status. Because eating behavior was not assessed in that study, the prevalence of impulsive eating or an ED in the sample remained unclear.

An important molecular mechanism for the mediation of G × E is the epigenetic regulation of gene expression, which might therefore be an interesting target to better understand EDs. The most intensely studied epigenetic mechanism is DNA methylation, the covalent addition of a methyl group to the 5’ position of a cytosine, primarily in the context of a cytosine-phosphate-guanine (CpG) dinucleotide. DNA methylation is most prevalent in regulatory regions of the genome including the gene promoter where it is in most, but not all cases negatively associated with transcription of the respective gene [20, 21]. In contrary, DNA methylation within the gene body is mostly, but also not exclusively, positively associated with gene transcription [22].

However, there is only a limited amount of literature on potential epigenetic changes associated with EDs, with most studies focusing on AN [11, 13, 19, 23–27]. Interestingly, two recent studies reported differential levels of DNA methylation in several sites of the *OXTR* gene in female patients with AN as compared to females remitted from AN and healthy females, and DNA methylation was correlated with markers of AN severity [13, 26]. However, only two studies so far investigated epigenetic alterations in patients with binge eating, but the *OXTR* was not included, and samples were heterogeneous [28, 29] and small (< 30 cases) [29].

Therefore, the aim of our study was to fill this research gap by investigating potential epigenetic differences in the *OXTR* in individuals on the obesity spectrum with BED (BED +) versus without BED (BED −) in order to further elucidate the role of OXT in impulsive eating behavior. We hypothesized that (a) DNA methylation of the *OXTR* will differ between individuals with BED (BED +) and without BED (BED −) and (b) methylation will be associated with self-reported eating behavior. To the best of our knowledge, previous studies in healthy participants without EDs have demonstrated effects of OXT administration on appetite regulation and eating behavior exclusively in male participants [15, 30–33]. Moreover, sex dependency of *OXTR* DNA methylation has been described earlier [34, 35]. Therefore, we additionally performed exploratory analyses of potential sex differences in *OXTR* methylation.

## Results

### Participants

Our sample comprised 183 females (BED+: *N* = 111, BED−: *N* = 72) and 44 males (BED+: *N* = 19, BED−: *N* = 25). Table 1 displays that both groups were comparable in terms of age and BMI; however, there was a significant difference in sex distribution with more females in the BED+ group. Regarding the *OXTR* rs53567 genotype, there was no significant difference between BED+ and BED− individuals. Data from the EDE-Q show that overall, the BED+ group reported significantly more severe eating pathology than the BED− group (Table 1).

**Table 1:** Sample characteristics regarding demographic characteristics, genotype and eating disorder psychopathology

	BED+	BED−	Group difference
N	130	97	–
Females/males (%)	85.4/14.6	74.2/25.8	*X*^*2*^ (1, *N* = 227) = 4.426, *p* = 0.035
Age (yrs), M ± SD^a^	41.0 ± 12.6	39.5 ± 13.6	T (1,217) = − 0.81, *p* = 0.42
Age (yrs), range	19 – 67	19 − 73	–
BMI (kg/m^2^), M ± SD^b^	38.7 ± 7.9	38.4 ± 6.5	T (1,222) = − 0.29, *p* = 0.77
*Genotype (%)*^*c*^			
Homozygous A/A	13.1	8.3	*X*^2^ (2, N = 227) = 2.17, *p* = 0.34
Heterozygous A/G	40.8	37.1	
Homozygous G/G	46.2	54.6	
*EDE-Q*^*d*^			
Restraint, M ± SD	1.7 ± 1.2	1.5 ± 1.5	T (1, 155) = − 0.996, *p* = 0.321
Eating Concern, M ± SD	2.3 ± 1.3	1.6 ± 1.2	T (1,155) = − 3.161, *p* = 0.002
Weight Concern, M ± SD	3.0 ± 1.4	1.8 ± 1.9	T (1,155) = − 4.317, *p* < 0.001
Shape Concern, M ± SD	3.8 ± 1.2	3.0 ± 1.8	T (1,155) = − 3.397, *p* = 0.001
Total Score, M ± SD	2.7 ± 0.9	2.0 ± 1.2	T (1,155) = − 4.370, *p* < 0.001

*BED+ *individuals with obesity and binge eating disorder; *BED− *individuals with obesity without binge eating disorder; *BMI* body mass index; and *EDE-Q* Eating Disorder Examination Questionnaire

^a^Data available for *n* = 219

^b^Data available for *n* = 224

^c^Percent values for genotype are calculated for BED+ and BED− individually

^d^Data available for *n* = 157

### Epigenetic analyses

**Fig. 1:**
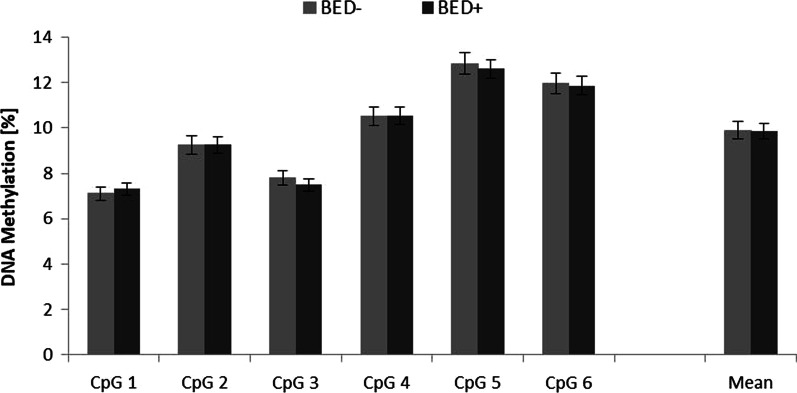
*OXTR* DNA methylation for all CpG sites analyzed (1-6) and for the average value across all 6 sites. BED− and BED+ patients were matched according to age and BMI. Values are displayed as means ± SEM

The Shapiro–Wilk test was used to assess mean DNA methylation distribution and showed that it departed significantly from normality (W(227) = 0.14, *p* =  < 0.001). The DNA methylation values of the six individual CpG sites within the *OXTR* as well as the mean across all sites were not significantly different between BED+ and BED− subgroups when analyzing the entire sample consisting of male and female individuals (Cohen’s *d* = 3.91 for the entire group) (Table 2 and Fig. 1).

**Table 2:** DNA methylation values for the individual CpG sites for each study group

CpG site	BED+ (%)	BED− (%)	Statistics
1	7.3 (± 0.28)	7.1 (± 0.29)	U = 6775.0; *p* = 0.337
2	9.26 (± 0.35)	9.24 (± 0.4)	*U* = 6440.0; *p* = 0.783
3	7.5 (± 0.27)	7.8 (± 0.31)	*U* = 5828.0; *p* = 0.330
4	10.53 (± 0.39)	10.5 (± 0.42)	*U* = 6408.0; *p* = 0.833
5	12.59 (± 0.39)	12.82 (± 0.47)	*U* = 6326.0; *p* = 0.966
6	11.87 (± 0.41)	12 (± 0.45)	*U* = 6298.0; *p* = 0.989
All	9.84 (± 0.34)	9.9 (± 0.37)	*U* = 6374.0; *p* = 0.888

*BED+ *individuals with obesity and binge eating disorder; *BED− *individuals with obesity without binge eating disorder

The DNA methylation values of all individual sites were in both groups (BED+ and BED −) strongly correlated with the mean across all sites analyzed (*r* ≥ 0.824; *p* < 0.001); therefore, subsequent analyses have been conducted using the mean DNA methylation value only.

In the total sample, a correlation could neither be detected between mean DNA methylation and BMI (*r*_part_ =  − 0.024; *p* = 0.767) nor EDE-Q total score (*r*_part_ =  − 0.069; *p* = 0.392) or any of the EDE-Q subscale scores (*r*_part_ < 0.108, *p* > 0.178).

**Fig. 2:**
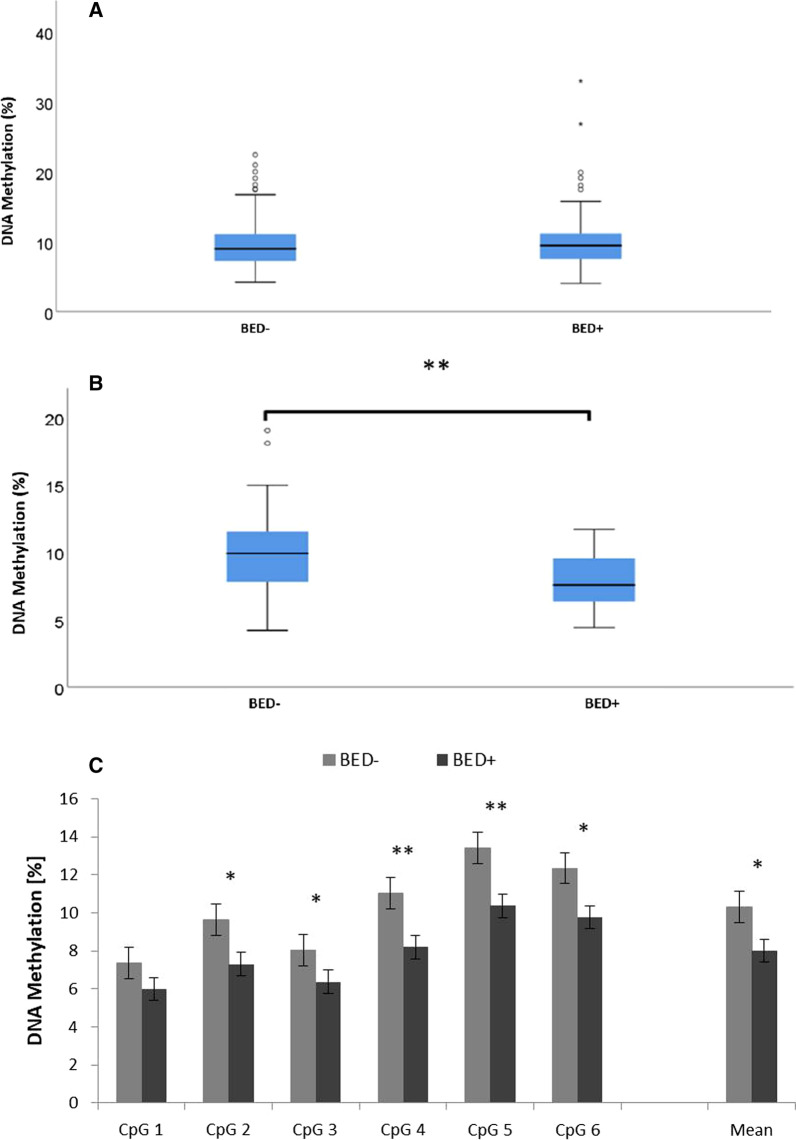
*OXTR* DNA methylation analyzed separately in females and males. Average DNA methylation in females (**A**) and males (**B**) with obesity without BED (BED** −**) versus with BED (BED +) as well as individual *OXTR* DNA methylation levels for CpG sites 1-6 is displayed (**C**). Values are displayed as means ± SEM

We additionally analyzed potential sex differences in *OXTR* methylation. ANOVA revealed no significant main effect of sex (F(1, 223) = 1.603; *p* = 0.207; *ƞ*_p_^2^ = 0.007) nor of group (F(1, 223) = 2.221; *p* = 0.138; *ƞ*_p_^2^ = 0.01). However, there was a significant sex × group interaction (F(1, 273) = 4.450; *p* = 0.036; *ƞ*_p_^2^ = 0.02). Post hoc subgroup analyses showed that *OXTR* DNA methylation was not significantly different between females of the BED+ group (10.16% ± 0.38%) and females of the BED− group (9.76% ± 0.44%; *U* = 4405.0; *p* = 0.243; Cohen’s *d* = 3.91; Fig. 2a), but males of the BED+ group displayed a significantly lower *OXTR* DNA methylation (7.99% ± 0.5%) than males of the BED− group (10.3% ± 0.72%; *U* = 141.0; *p* = 0.022; Cohen’s *d* = 3.06; Fig. 2b). Post hoc analyses including individual CpG sites revealed that %DNA methylation at CpG site 2-6 was significantly different between BED+ and BED− males, whereas %DNA methylation at CpG 1 site did not differ between groups (Table 3, Fig. 2c). *OXTR* rs53567 genotype was in neither group associated with DNA methylation levels (data not shown).

**Table 3:** DNA methylation values for the individual CpG sites in males of each study group

CpG site	BED+ (%)	BED− (%)	Statistics
1	5.99 (± 0.50)	7.34 (± 0.53)	U = 162.0; *p* = 0.074
2	7.29 (± 0.54)	9.62 (± 076)	*U* = 147.0; *p* = 0.032
3	6.37 (± 0.42)	8.05 (± 0.60)	*U* = 151.5; *p* = 0.042
4	8.19 (± 0.50)	11.03 (± 0.78)	*U* = 129.0; *p* = 0.010
5	10.36 (± 0.59)	13.40 (± 0.85)	*U* = 132.0; *p* = 0.012
6	9.76 (± 0.60)	12.35 (± 0.85)	*U* = 142.0; *p* = 0.024
All	8.00 (± 0.50)	10.30 (± 0.72)	*U* = 141.0; *p* = 0.022

*BED+ *individuals with obesity and binge eating disorder; *BED− *individuals with obesity without binge eating disorder

Genotype distribution did neither differ between BED+ and BED− females (*X*^2^ (2), *N* = 183) = 2.89, *p* = 0.24) nor BED+ and BED− males (*X*^2^ (2), *N* = 44) = 1.6, *p* = 0.45).

## Discussion

In the present study, we investigated potential epigenetic differences in the *OXTR* gene in a sample of individuals with obesity with and without a comorbid BED in order to further elucidate the potential contribution of the OXT system to disordered eating behavior and body weight regulation.

Overall, we did not find between-group differences in *OXTR* methylation when analyzing the complete sample. However, subgroup analyses of females and males revealed that male patients with obesity and BED (BED +) showed a significantly lower *OXTR* DNA methylation than male individuals with obesity but without BED (BED −) of comparable age and BMI. No such difference was found in the female subsample. Hence, our first hypothesis regarding group differences in DNA methylation of the *OXTR* gene was partly supported. We additionally performed correlational analyses to investigate our second hypothesis on associations between methylation and indicators of eating behavior, but found no relationship between mean DNA methylation and BMI nor with self-reported ED pathology according to EDE-Q. Hence, our second hypothesis was not supported.

It is not straightforward to interpret the implications of these epigenetic findings differentiating male individuals with and without BED without functional data. In a previous study, high levels of DNA methylation within the analyzed CpG island of the *OXTR* gene were found to downregulate gene expression [36]. However, as *OXTR* gene expression was not analyzed in our study, we can only speculate whether the observed methylation differences might be associated with an overexpression of *OXTR*. On the other hand, there is ongoing controversy on the relationships between *OXTR* concentration, body weight regulation and eating behavior. When comparing circulating OXT concentrations between individuals with obesity and normal weight, higher [37], unchanged [38] or decreased [39] concentrations have been reported for obesity. This inconsistent evidence might partly be due to sampling approaches, but also due to heterogeneity of investigated samples which were partly characterized by somatic comorbidities [37]. Our study adds to this discussion by showing that male individuals with obesity also differ in the OXT system on grounds of a comorbid ED, pointing toward a complex interplay of OXT signaling, body weight regulation and eating behavior. Based on a data set across the whole spectrum of body weight, Schorr et al. [37] have recently suggested that OXT is a ‘marker of energy availability’ and that obesity represents a particular OXT-sensitive state. The latter assumption is supported by findings that acute OXT administration results in a stronger decrease in food intake in males with obesity as compared to males with normal weight [15], a pattern previously suggested to reflect a compensatory response of a OXT-sensitive system [37]. Differential regulation of DNA methylation of the *OXTR* gene might contribute to this OXT-sensitive state in obesity, and this might be of particular relevance for the subgroup of individuals with BED showing impulsive eating behavior. In this context, it is also important to consider that the anorexigenic effect of OXT might be partly communicated via effects on reward-system functioning as well as cognitive control circuits [9, 10, 31, 40], and both of these regulatory systems are supposed to play a pivotal role in BED and partly differentiate obese individuals with from those without BED [5, 6, 41]. Interestingly, a recent study showed that a single dose of intranasal OXT improves performance on an inhibitory control task in males with overweight and obesity [31]. Differential DNA gene methylation might play a role in OXT signaling along this cognitive control pathway, especially in the highly impulsive phenotype which is represented by BED on the obesity spectrum.

The observed sex difference in *OXTR* methylation could be explained with different functions of OXT in females versus males, for instance, as it has been described for the regulation of anxiety-related and social behaviors [42]. Besides, OXT generally plays a prominent role in the female organism. Sex differences in the role of OXT in the regulation of eating behavior have previously been described in animal models [43]. In line with the specificity of our results for males, only male mice with OXT deficiency due to ablated OXT neurons were more likely to develop obesity when put on a high-fat diet [43]. However, there is up to now a scarcity on data on sex differences in OXT function associated with the regulation of eating behavior [40], and it has been emphasized that taking sex context into account is warranted [10].

Regarding our second hypothesis, two previous epigenetic studies performed in samples of patients with AN reported correlations of OXTR methylation with measures of illness severity as well as measures assessing attachment and social behavior [13, 26]. The lack of findings in our sample could be due to the variables used, i.e., although representing an objective measure, BMI is only a very rough indicator of eating behavior, and regarding more behavioral constructs, it could be that measures that are more closely related to impulsive aspects of eating would have yielded different results. It is also possible that other measures associated with OXT functions related to attachment, stress or anxiety would have been insightful. As the anorexigenic effects of OXT might partly be communicated by influencing reward-related brain circuits and inhibitory control networks [10, 31], it will be important for future studies to investigate interactions of OXT function and dimensions of impulsivity versus cognitive control to understand the underlying mechanisms especially in the spectrum of under controlled eating behavior and along the obesity spectrum. Two recent studies suggest that there might be a specific and positive influence of OXT administration on inhibitory control in men on the obesity spectrum [31] and in women characterized by impulsive eating behavior [44]. This might also translate to food intake, but a recent study in women with BED could not find any effects of a single dose of OXT on eating behavior [45]. As discussed above, this could partly be due to sex differences in OXT functioning.

Finally, we did not detect an effect of the *OXTR* variant rs53567 in our sample. This is not overly surprising because our study, although of decent sample size with regard to epigenetic analyses, might have been underpowered to detect genetic associations [46].

### Strengths and limitations

Evidence on potential epigenetic changes associated with EDs is still very limited and there is a specific lack of studies in BED [24]. Our data stem from a well-characterized treatment-seeking sample with severe ED pathology, a high BMI and a confirmed ED diagnosis, and we have compared the BED group with a treatment-seeking sample of age- and BMI-matched patients with obesity who were not diagnosed with ED. This offers the opportunity to gain more knowledge on phenotypes within the heterogeneous spectrum of obesity, which points toward more individualized treatment targets for sub-populations. Our sample size of N > 200 is decent compared to most previous studies, but still might be underpowered to detect more subtle differences [24]. BED diagnoses were based on DSM-IV criteria allowing for a consistent diagnostic approach; notably, the BED criteria according to DSM-IV as compared to DSM-5 are narrower due to a more conservative time criterion [3, 47]. However, limitations of the study comprise that we did not assess and control for some variables known to potentially confound epigenetic profiles, including medication, smoking and socioeconomic status [18, 24]; that we did not include a self-report instrument assessing impulsivity; that we have no information about mental comorbidities; and that we had missing self-report data in the sample. It should also be noted that the male subgroup of our sample—in which significant findings regarding BED+/BED− and DNA methylation were detected—was smaller than the female subgroup. Furthermore, it would have been very interesting to investigate DNA methylation differences between individuals with and, respectively, without BED on an epigenome-wide scale in order to gain more insight into the dysregulation of epigenetic processes underlying BED. However, due to the moderate sample size, we decided to perform a targeted analysis rather than a hypothesis-free screening.

Further studies, that also assess OXTR expression and OXT directly, are needed to fully unravel the contribution of the OXT system, and dysfunctions thereof, to the spectrum of poorly controlled eating behavior and overweight. It is a limitation of the present study that these mechanistic questions remain unanswered as we did not collect RNA and serum samples. Moreover, DNA methylation patterns are tissue- and cell type-specific [48], and results obtained in blood samples may not directly reflect DNA methylation alterations in the brain [49], i.e., the decisive organ in the control of eating behavior (which is generally not available for tissue molecular analyses in living individuals). The CpG sites we investigated in our study are not represented on the Illumina 450 K array and are therefore not included in common online tools designed for the comparison of DNA methylation patterns between blood and brain (e.g., BECon [50]). However, correlations between other intragenic OXTR CpG sites represented in BECon are variable (reaching, e.g., values of 0.67 for cg27501759 in blood vs. BA10), so that we cannot draw direct conclusions on whether our results observed in the peripheral blood of male individuals with BED are mirrored by changes within the brain. Nevertheless, OXTR DNA methylation in peripheral blood may still be a useful biomarker to identify different subtypes of BED+ individuals.

Future studies should ideally also include control groups of normal-weight individuals with or without BED, and they should also shed light on the question why OXTR methylation is impaired in males with BED, e.g., if this pattern is caused by impaired DNA methyltransferase activity and specific for BED. In addition, as DNA methylation is a reversible epigenetic signal, it would be a promising avenue to investigate reversibility of the observed OXTR DNA methylation in the context of a therapeutic intervention such as psychotherapy in patients with BED. Furthermore, effects of intranasal OXT administration on OXTR DNA methylation should be addressed in order to gain insight into potential mechanisms underlying the observed differential OXTR DNA methylation in male patients with BED. Previous studies using intranasal OXT administration have been performed partly in male and female samples, and some of them have demonstrated effects on inhibitory control [31, 44]. The investigation of putative interactions of intranasal OXTR administration with different epigenetic profiles would be an interesting next step and may inform tailored interventions.

## Conclusions

Our data from an epigenetic analysis of DNA methylation of the *OXTR* gene in the obesity spectrum suggest that clinically significant binge eating pathology is associated with lower *OXTR* DNA methylation exclusively in males. This supports previous evidence that OXT (dys)function might play a role in impulsive eating behavior, potentially predominantly in males. Additionally, the data support the view that individuals with BED represent a distinct phenotype within the obesity spectrum that is characterized by specific vulnerability factors, potentially also depending on the respective sex [5, 6]. The sex differences in *OXTR* DNA methylation pose further questions concerning the complex influence of OXT on the regulation of eating behavior in females versus males that has gained little attention so far [10]. The OXT system has been identified and piloted as a potential treatment target in eating and weight disorders, especially for those EDs associated with impulsive eating patterns [3, 9, 11, 51], as short-term intranasal administration of OXT has anorexigenic effects [52]. A better data basis on genetic and epigenetic underpinnings of the OXT system in ED populations will also contribute to this interventional field of research as it may reveal how and for whom OXT administration might work best as a potential adjunctive treatment.

## Methods

### Sample description

We investigated DNA methylation in a group of 227 individuals with obesity. Participants were recruited between 2014 and 2020 from the inpatient, day-patient and outpatient services of the Department of Psychosomatic Medicine and Psychotherapy at the University Hospital Tübingen. Inclusion criteria for study participation were a diagnosis of obesity or BED and providing written informed consent; no specific exclusion criteria applied. All participants underwent a structured and standardized diagnostic assessment in which, among others, height and weight were assessed by study staff in order to calculate BMI, a structured clinical examination was conducted in order to assess or exclude a potential eating disorder diagnosis according to DSM-IV [47], self-reported eating disorder pathology was assessed using the EDE-Q [53], and ethylenediaminetetraacetic acid (EDTA) peripheral venous blood samples were collected and kept at − 80 °C until further usage. For the epigenetic analysis, we compared two subgroups of the sample: participants with obesity and excluded comorbid BED (BED −) and participants with obesity and a diagnosed BED (BED +). All participants provided written informed consent. The study was approved by the ethics committee of the Medical Faculty of the Eberhard Karls University Tübingen, Germany (218/2018BO2).

### Epigenetic analyses

Extraction of genomic DNA from EDTA anti-coagulated venous blood for genetic and epigenetic analysis was performed using the QIAamp DNA Blood Maxi-Kit (Qiagen, Hilden, Germany). The analyzed region lies within the CpG island spanning exons 1 to 3 (Chr3:8 808 962–8 811 280, GRCh37/hg19) [54] of the OXTR gene. This locus has been investigated with regard to several psychiatric phenotypes [55–58] and has provided evidence for a negative association between DNA methylation and gene expression (36, 55). Due to the technical limitations of our pyrosequencing approach which enables the analysis of only short reads with good quality, we further narrowed down our sequence of interest to (Chr3:8 809,414–8 809,444, GRCh37/hg19) in order to include the sequence analyzed by Ziegler et al. [57] in the context of social anxiety and by Schiele et al. [58] in the context of obsessive–compulsive disorder. SNP rs53576 is located in the intronic region downstream of the analyzed region. 500 ng genomic DNA was bisulfite converted using the EpiTect Fast Bisulfite Conversion Kit (Qiagen), and the region of interest within the *OXTR* gene was amplified using the PyroMark PCR Kit (Qiagen) according to the manufacturer’s instructions. PCR and sequencing primer (Metabion, Planegg, Germany) were as follows: PCR forward primer: 5′-TGTTGTTGTTTATGTTTTTGGAT-′3, PCR reverse primer: Biotin-5′- -CCTTAAATCCCCAAAACTAAAT-′3, sequencing primer: 5′-GTTTGGTTATTTGTTAG-3′.

Successful amplification as well as specificity of the PCR products was verified via agarose gel electrophoresis. Several PCR runs were performed as technical replicates for each sample (minimum two replications). Processing of the PCR amplicons for pyrosequencing analysis was performed according to the manufacturer’s protocol, and PCR products were then sequenced using the PyroMark Q24 system and the PyroMark GoldReagents (Qiagen). The level of methylation in every sample was quantified using the PyroMark Q24 software version 2.0.6 (Qiagen). The pyrosequencing assay contained six CpG sites. Only samples with standard deviation of < 3% between technical replicates were included in the analysis. To detect potentially biased amplification of differentially methylated fragments, DNA samples with known methylation levels (0%, 25%, 50%, 75% and 100%) were included as controls (EpiTect Control DNA, Qiagen) in the amplification and the pyrosequencing analysis. In all steps of the DNA methylation analysis (bisulfite conversion, PCR and pyrosequencing), samples were processed in balanced design in order to avoid batch effects.

### Genotyping

*OXTR* rs53567 was genotyped on a StepOne system (ThermoFisher Scientific; Waltham; USA) using TaqMan® SNP Genotyping Assay (ThermoFisher Scientific) and the standard protocol for allelic discrimination. Accuracy was assessed by duplicating 15% of the original sample, and reproducibility was 100%. The genotype frequencies did not deviate from Hardy–Weinberg equilibrium (HWE; *p* = 0.69).

### Statistical analyses

An a priori power analyses using GPower 3.1 [59] and calculating an ANOVA (fixed-effects, omnibus, one-way) revealed that a sample size of 180 individuals in total would be sufficient to detect a significant difference between the investigated groups (BED+ versus BED −) with a power of 0.80 assuming a moderate effect size of *f* = 0.25. Therefore, with our sample of 227 individuals we would be able to detect a moderate effect of *f* = 0.22 with a power of 0.80.

Statistical analyses were performed using SPSS Statistics Version 26 (IBM, NY, USA). The Shapiro–Wilk test was used to assess data distribution. Independent sample *t* tests were used to compute group differences in the sample characteristics. Mann–Whitney U test was used to compute group differences in *OXTR* DNA methylation between BED− and BED+ individuals. Chi-squared tests were used to analyze the distribution of genotypes for each group. A 2 × 2 ANOVA was performed to investigate differences in *OXTR* DNA methylation between BED+ and BED− individuals with %DNA methylation as depended variable, group (BED+ vs. BED −) and sex as between-group factors. Differences in mean DNA methylation in the female and male subgroup were calculated using Mann–Whitney U tests. Correlation between DNA methylation levels of the individual sites (CpG 1-6) and the mean across all 6 sites was calculated using Spearman correlation. To analyze relationships between DNA methylation levels and eating behavior, we performed partial Pearson correlations with BMI and EDE-Q scores controlling for patient group. Results were considered significant when *p* ≤ 0.05. As *p* values for our two main hypotheses were > 0.05 and as analyses on potential sex differences were exploratory, no adjustment procedure for multiple testing was applied.

## Data Availability

The datasets used and/or analyzed during the current study are available from the corresponding author on reasonable request.
